# Targeted tumor dual mode CT/MR imaging using multifunctional polyethylenimine-entrapped gold nanoparticles loaded with gadolinium

**DOI:** 10.1080/10717544.2017.1422299

**Published:** 2018-01-04

**Authors:** Benqing Zhou, Zuogang Xiong, Peng Wang, Chen Peng, Mingwu Shen, Serge Mignani, Jean-Pierre Majoral, Xiangyang Shi

**Affiliations:** ^a^ Department of Radiology, Shanghai Tenth People’s Hospital, Tongji University School of Medicine Shanghai P. R. China; ^b^ State Key Laboratory for Modifcation of Chemical Fibers and Polymer Materials, College of Chemistry, Chemical Engineering and Biotechnology, Donghua University Shanghai P. R. China; ^c^ Laboratoire de Chimie et de Biochimie Pharmacologiques et Toxicologique, Université Paris Descartes, PRES Sorbonne Paris Cité Paris France; ^d^ CQM – Centro de Química da Madeira, MMRG, Universidade da Madeira Funchal Portugal; ^e^ Laboratoire de Chimie de Coordination du CNRS Toulouse France; ^f^ UPS, INPT, Université de Toulouse Toulouse France

**Keywords:** Polyethylenimine, gold nanoparticles, CT imaging, MR imaging, tumor targeting

## Abstract

We report the construction and characterization of polyethylenimine (PEI)-entrapped gold nanoparticles (AuNPs) chelated with gadolinium (Gd) ions for targeted dual mode tumor CT/MR imaging *in vivo.* In this work, polyethylene glycol (PEG) monomethyl ether-modified PEI was sequentially modified with Gd chelator and folic acid (FA)-linked PEG (FA-PEG) was used as a template to synthesize AuNPs, followed by Gd(III) chelation and acetylation of the remaining PEI surface amines. The formed FA-targeted PEI-entrapped AuNPs loaded with Gd (FA-Gd-Au PENPs) were well characterized in terms of structure, composition, morphology, and size distribution. We show that the FA-Gd-Au PENPs with an Au core size of 3.0 nm are water dispersible, colloidally stable, and noncytotoxic in a given concentration range. Thanks to the coexistence of Au and Gd elements within one nanoparticulate system, the FA-Gd-Au PENPs display a better X-ray attenuation property than clinical iodinated contrast agent (e.g. Omnipaque) and reasonable r_1_ relaxivity (1.1 mM^−1^s^−1^). These properties allow the FA-targeted particles to be used as an efficient nanoprobe for dual mode CT/MR imaging of tumors with excellent FA-mediated targeting specificity. With the demonstrated organ biocompatibility, the designed FA-Gd-Au PENPs may hold a great promise to be used as a nanoprobe for CT/MR dual mode imaging of different FA receptor-overexpressing tumors.

## Introduction

Imaging techniques have developed exponentially during the past decades and many techniques, such as magnetic resonance (MR) imaging (Chen et al., 2014; Li et al., 2015b), computed tomography (CT) (Liu et al., 2012; Yin et al., 2013), radionuclide imaging (Nahrendorf et al., 2008; Guo et al., 2016), and ultrasound imaging (Lee et al., 2015), etc. have become indispensable in clinical use. Nanoparticles (NPs) have been widely used in imaging techniques due to their unique electric, magnetic, and optical properties (Brigger et al., 2002; Laurent et al., 2008; Boisselier & Astruc, 2009). For example, a hypoxia-sensitive nitroimidazole-conjugated bovine serum albumin can be used to prepare AuNPs with a diameter of 3.7 nm for CT imaging of tumor hypoxia (Shi et al., 2016). Polyethylene glycol (PEG)-derivatized phosphine oxide can be used to cap Fe_2_O_3_ NPs (size = 3 nm) formed via thermal decomposition of iron-oleate complex in the presence of oleyl alcohol. The generated small sized Fe_2_O_3_ NPs displayed a good biocompatibility and high r_1_ relaxivity of 4.78 mM^−1^s^−1^ and could be used for high-resolution T_1_-weighted MR imaging of blood pool (Kim et al., 2011). Mesoporous silica NPs coated with AuNPs have been synthesized and used as a nanoprobe for ultrasound-induced cytoclasis, contrast-intensified ultrasound imaging, and ultrasound imaging-guided high intensity focused ultrasound surgical therapy (Wang et al., 2013).

In most cases, each imaging modality has its own intrinsic virtues and drawbacks. For instance, CT imaging as a structural imaging mode affords reconstruction of three-dimensional tomography with high spatial resolution, but falls behind with poor sensitivity for soft tissues (Lee et al., 2013). MR imaging exhibits high spatial resolution, noninvasiveness, and outstanding capacity to differentiate soft tissues, but has poor contrast sensitivity to discriminate tumor from the surrounding tissues (Veiseh et al., 2010). Therefore, it is essential to combine dual- or multi-modal imaging for further improvement of the diagnosis accuracy. For example, prostate-specific membrane antigen (PSMA)-targeted iron oxide NPs (diameter = 11 nm) were prepared using a one-pot method and used for targeted MR imaging of tumor *in vivo*. After labeling with gallium, the particles could also be used for targeted tumor positron emission tomography imaging (Moon et al., 2016). Dendrimer-entrapped AuNPs loaded with gadolinium (Gd) ions were synthesized and used for dual mode CT/MR imaging of blood pool and tumors (Chen et al., 2013; Wen et al., 2013a; Chen et al., 2015). Moreover, it is well-documented that tumor accumulation of NPs through enhanced permeability and retention (EPR) effect is less efficient than that through active targeting based on receptor-mediated endocytosis pathway (Gao et al., 2004; Liong et al., 2008; Wang & Thanou, 2010; Bertrand et al., 2014). Hence, targeting ligands of folic acid (FA) (Li et al., 2013; Hu et al., 2016), hyaluronic acid (Li et al., 2014; Hu et al., 2015b; Li et al., 2015a), lactobionic acid (Liu et al., 2014; Hu et al., 2017), or arginine-glycine-aspartic peptide (Chen et al., 2015; Hu et al., 2015a; Xu et al., 2017) have been linked onto various nanoplatforms to achieve active targeting.

Branched polyethylenimine (PEI) possessing good water solubility and abundant surface amines has been used as a powerful vehicle to coat NPs or encapsulate drugs (Appelhans et al., 2009; Hoebel et al., 2011; Cai et al., 2013; Chen et al., 2016; Kong et al., 2017; Zhou et al., 2017). In our previous work, we have shown that branched PEI can be used either as a stabilizer to form PEI-stabilized AuNPs (Tong et al., 2012; Wen et al., 2013b), or as a template to entrap AuNPs for blood pool/organ or tumor CT imaging (Zhou et al., 2014; Zhou et al., 2016a,b ). Particularly, we have shown that PEGylated PEI can be used to entrap AuNPs and load Gd(III) for dual mode CT/MR imaging of blood pool and major organs of mice (Zhou et al., 2016a). This prior work prompts us to hypothesize that targeting ligand can be modified onto the surface of the PEGylated PEI, thereby forming a multifunctional PEI-based nanoplatform for targeted tumor CT/MR imaging.

**Figure 1.**
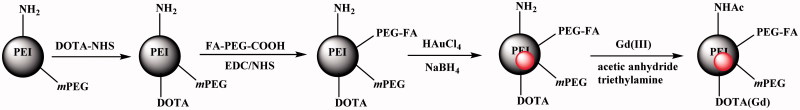
Schematic illustration of the preparation of the FA-Gd-Au PENPs.

In this work, PEG monomethyl ether-modified PEI was sequentially modified with Gd(III) chelator (DOTA) and PEGylated FA and was used as a template to synthesize AuNPs, followed by Gd(III) chelation and complete acetylation of the remaining PEI surface amines (Figure 1). The formed FA-targeted PEI-entrapped AuNPs loaded with Gd (FA-Gd-Au PENPs) were well characterized via different techniques. The cytocompatibility of the particles was assessed by cell viability assay and cell morphology observation. The targeting specificity of the particles was tested by cellular Au uptake assay via inductively coupled plasma-optical emission spectroscopy (ICP-OES) and confocal microscopy. The potential to use the FA-Gd-Au PENPs for CT/MR imaging was explored by CT/MR phantom studies and CT/MR imaging of a xenografted tumor model *in vivo*.

## Materials and methods

### Materials

Branched PEI (Mw = 25,000), 1-ethyl-3-(3-dimethylaminopropy) carbodiimide hydrochloride (EDC), and N-hydroxysuccinimide (NHS) were obtained from Sigma-Aldrich (St. Louis, MO). PEG monomethyl ether with the other end of carboxyl group (mPEG-COOH, Mw = 2000) and dual functional PEG with one end of carboxyl group and the other end of amine group (NH_2_-PEG-COOH, Mw = 2000) were acquired from Shanghai Yanyi Biotechnology Corporation (Shanghai, China). FA was supplied by J&K Chemical Ltd. (Shanghai, China). 2,2’,2’’-(10-(2-(2,5-Dioxopyrrolidin-1-yloxy)-2-oxoethyl)-1,4,7,10-tetraazacyclododecane-1,4,7-triyl) triacetic acid (DOTA-NHS) was from CheMatech (Dijon, France). Dimethyl sulfoxide (DMSO), acetic anhydride, triethylamine, gold chloride (HAuCl_4_·4H_2_O), and all the other chemicals and solvents were obtained from Sinopharm Chemical Reagent Co., Ltd. (Shanghai, China). 3-(4,5-Dimethylthiazol-2-yl)-2,5-diphenyltetrazolium bromide (MTT) was acquired from Shanghai Sangon Biological Engineering Technology & Services Co., Ltd. (Shanghai, China). HeLa cells (a human cervical carcinoma cell line) were from Institute of Biochemistry and Cell Biology (the Chinese Academy of Sciences, Shanghai, China). Dulbecco’s modified Eagle medium (DMEM), fetal bovine serum (FBS), penicillin, and streptomycin were purchased from HyClone Lab., Inc. (Logan, UT). All chemicals and materials were used as received. Water used in all the experiments was purified using a Milli Q Plus 185 water purification system (Millipore, Bedford, MA) with a resistivity greater than 18.2 MΩ·cm. Regenerated cellulose dialysis membranes with a molecular weight cutoff (MWCO) of 1000 or 14,000 were acquired from Fisher (Pittsburgh, PA).

### Synthesis of the FA-Gd-Au PENPs

Folic Acid-PEG-Acid (FA-PEG-COOH) and PEI-mPEG were prepared according to the literature (Zhou et al., 2016b). The formed PEI-mPEG was next linked with DOTA. In brief, DOTA (3.76 mg, 5 mL DMSO) was added into the PEI-mPEG solution (10 mg, 5 mL DMSO) under stirring for one day, and then EDC-activated FA-PEG-COOH (11.4 mg, 5 mL DMSO) was added to the above mixture solution under stirring for three days. The reaction mixture was dialyzed and lyophilized according to literature processes (Zhou et al., 2016a) to get the product of PEI-mPEG-DOTA-(PEG-FA).

The formed PEI-mPEG-DOTA-(PEG-FA) was used as a template to entrap AuNPs, followed by Gd(III) ions chelation and acetylation of the remaining PEI surface amines. In short, HAuCl_4_ (10 mg/mL, in 2.81 mL water) was added dropwise into the PEI-mPEG-DOTA-(PEG-FA) water solution (25 mg, 100 mL) under vigorous stirring for 15 min and then sodium borohydride (12.95 mg, in 5 mL icy water) with five times molar excess to the Au salt was quickly added to the above mixture under stirring for 3 h. Hereafter, 5 mL of gadolinium(III) nitrate hexahydrate (Gd(NO_3_)_3_·6H_2_O; 4.65 mg) was added into the above mixture and reacted for 24 h to get the raw product of {(Au^0^)_200_-PEI-*m*PEG-DOTA(Gd)-(PEG-FA)} PENPs. Lastly, the raw product was acetylated and purified according to the literature (Zhou et al., 2014) to get the final product of {(Au^0^)_200_-PEI-NHAc-*m*PEG-DOTA(Gd)-(PEG-FA)} PENPs (for short, FA-Gd-Au PENPs). For comparison, the non-targeted {(Au^0^)_200_-PEI-NHAc-*m*PEG-DOTA(Gd)} PENPs were also prepared and characterized according to our previous work (Zhou et al., 2016a).

### Characterization techniques

^1 ^H NMR spectra were recorded on a Bruker AV400 nuclear magnetic resonance spectrometer (Karlsruhe, Germany). Samples were dissolved in heavy water (D_2_O) before measurements. Ultraviolet-visible (UV-Vis) spectroscopy was carried out using a Lambda 25 UV-Vis spectrophotometer (PerkinElmer, Waltham, MA). TEM was performed using a JEOL 2010 F analytical electron microscope (JEOL, Tokyo, Japan) operating at 200 kV. TEM samples were prepared by depositing a sample suspension (0.1 mg/mL) onto carbon-coated copper grid and air dried before measurements. Dynamic light scattering (DLS) and zeta potential measurements were conducted using a Malvern Zetasizer Nano ZS model ZEN3600 (Worcestershire, UK) with a standard 633 nm laser. Samples were dissolved in water at a concentration of 0.5 mg/mL before measurements.

### Cytotoxicity evaluation of the FA-Gd-Au PENPs

Cytotoxicity of the FA-Gd-Au PENPs was evaluated by MTT cell viability assay. In brief, 1 × 10^4^ HeLa cells were seeded in each well of a 96-well plate with 200 μL of DMEM. After 24 h incubation, the medium was replaced with fresh medium containing FA-Gd-Au PENPs at different Au concentrations (0–100 μM) and the cells were incubated for another 24 h. Then, MTT (20 μL, 5 mg/mL) was added to each well and the cells were incubated for another 4 h. Later, the medium was carefully removed and 200 μL of DMSO was added into each well to dissolve the formed formazan crystals. The optical density value at 570 nm in each well was measured by a Thermo Scientific Multiskan MK 3 ELISA reader (Thermo Scientific, Waltham, MA). Mean and standard deviation (SD) were reported based on the triplicate wells for each sample.

Likewise, after HeLa cells were treated with the FA-Gd-Au PENPs at different Au concentrations (0–100 μM) for 24 h, the morphology of cells was observed using a Leica DM IL LED inverted phase contrast microscope (Wetzlar, Germany) with a magnification of 100 × for each sample.

### Cellular uptake assays

HeLa cells were separately cultured in a six-well plate at a density of 1 × 10^6^ cells per well the day before the experiment to bring the cells to confluence. The medium was replaced with fresh medium containing FA-Gd-Au PENPs or nontargeted Gd-Au PENPs with different Au concentrations ([Au] = 0–300 μM) and the cells were incubated for another 3 h. Then, the cells were washed three times with PBS and were digested using aqua regia solution before quantification by Leeman Prodigy ICP-OES (Hudson, NH).

Cellular uptake of the particles was further evaluated by confocal microscopic observation using a Carl Zesis LSM 700 laser scanning confocal microscope (Jena, Germany). The cells were plated onto coverslips with a diameter of 14 mm pretreated with 5% hydrochloric acid, 30% nitric acid, and 65% alcohol in the wells of 24-well plate. After incubation with the FA-Gd-Au PENPs or nontargeted Gd-Au PENPs ([Au] = 100 μM) for 12 h, the cells were washed with PBS for three times and were fixed with glutaraldehyde (2.5%) for 15 min at 4 °C. The cell nuclei were then counterstained with 4',6-diamidino-2-phenylindole (DAPI, BestBio, Shanghai, China) for 7 min at 37 °C. The samples were scanned using a 63 × oil immersion objective lens.

### CT/MR phantom studies

Water solutions of FA-Gd-Au PENPs and Omnipaque (as a control) with different Au or iodine concentrations (0.005, 0.01, 0.02, 0.06, or 0.1 M, respectively) were prepared in 1.5 mL-Eppendorf tubes (100 μL in each tube) and placed in a homemade scanning holder. CT phantom studies were performed using a dual-source CT system (SOMATOM Definition Flash, Siemens, Erlangen, Germany) with 80 kV, 110 mA, and a thickness of 0.6 mm.

For MR imaging, the FA-Gd-Au PENPs with different Gd concentrations in water solution (0.0625, 0.125, 0.25, 0.5, or 1 mM, respectively) were measured by a 0.5-T NMI20-Analyst NMR analyzing and imaging system (Shanghai Niumag Corporation, Shanghai, China). T_1_-weighted images were recorded using a spin-echo imaging sequence with the following parameters: point resolution = 156 mm ×156 mm, slice thickness = 0.6 mm, TR = 4000 ms, TE = 60 ms, and number of excitations = 1. The r_1_ relaxivity was calculated by linearly fitting the inverse T_1_ relaxation time (1/T_1_) as a function of Gd concentration.

### Targeted CT and MR imaging of a xenografted tumor model *in vivo*

Animal experiments were strictly performed according to protocols approved by the institutional committee for animal care and also in accordance with the policy of the National Ministry of Health. BALB/c nude mice and C57BL/6 mice (both 4–6 weeks old) were provided by Shanghai SLAC Laboratory Animal Center (Shanghai, China). The BALB/c nude mice were injected subcutaneously with 1.5 × 10^6^ cells/mouse on the right side of their backs. Until the tumor nodules reached a volume of 200–300 mm^3^, the nude mice were anesthetized by pentobarbital sodium (40 mg/kg). For *in vivo* CT imaging, the tumor-bearing nude mice were intravenously injected with the FA-Gd-Au PENPs or nontargeted Gd-Au PENPs ([Au] = 0.1 M, in 150 μL PBS for each mouse) and scanned before and at different time points post-injection using the same scanning parameters as described above.

For *in vivo* MR imaging, the tumor-bearing nude mice were intravenously injected with the FA-Gd-Au PENPs or nontargeted Gd-Au PENPs ([Gd] = 0.01 M, in 150 μL PBS for each mouse) and scanned before and at different time points post-injection. The *in vivo* MR images were acquired using a 3 T clinical MR imaging instrument (SOMATON Definition Flash, Siemens, Erlangen, Germany) with the following parameters: TR = 280 ms, TE = 15 ms, FOV = 80 × 100, matrix = 318 × 314, thickness = 0.7 mm, and gap = 0.14 nm. The MR signal enhancement percentage was calculated by dividing the MR signal/noise ratio (SNR) of tumor before injection with that of tumor after injection.

### *In vivo* biodistribution and hematoxylin and eosin (H&E) staining of major organs of mice

The tumor-bearing nude mice were intravenously injected with the FA-Gd-Au PENPs ([Au] = 0.1 M, in 150 μL PBS for each mouse) and sacrificed at different time points post-injection. Then their heart, liver, spleen, lung, and kidney together with tumor were extracted, weighed, cut into small pieces, and then digested overnight before being measured by ICP-OES.

C57BL/6 mice were intravenously administrated with the FA-Gd-Au PENPs ([Au] = 0.1 M, in 100 μL PBS for each mouse) or 100 μL PBS (as a control). After one month treatment, the mice were anesthetized and sacrificed, and their major organs were harvested. These organs were then fixed, embedded, sectioned, and stained with H&E using a standard procedure (Peng et al., 2013) before optical microscopic observation.

### Statistical analysis

One way analysis of variance (ANOVA) statistical analysis method was used to assess the significance of the experimental data. A value of 0.05 was selected as the significance level and the data were marked as (*) for *p* < .05, (**) for *p* < .01, and (***) for *p* < .001, respectively.

## Results and discussion

### Preparation and characterization of the FA-Gd-Au PENPs

In this experiment, PEI was sequentially modified with mPEG-COOH, DOTA, and FA-PEG-COOH. The prepared multifunctional PEI-mPEG-DOTA-(PEG-FA) was used as a template to entrap AuNPs, followed by Gd(III) chelation and full acetylation of the remaining PEI surface amines (Figure 1). The designed FA-Gd-Au PENPs were well characterized via different methods.

^1 ^H NMR was used to characterize the formed FA-PEG-COOH segments and the DOTA and PEG conjugation on the branched PEI platform. The peak at 3.4–3.6 ppm is attributed to methylene protons of PEG and those at 6.5–8.8 ppm is associated to the FA protons. The average number of FA moieties conjugated onto each PEG was measured to be 0.7 according to the NMR integration (Supplementary Figure S1(a)). Similarly, through NMR integration, the average numbers of FA, DOTA (δ: 3.0–3.5 ppm) and PEG moieties conjugated onto each PEI (δ: 2.2–2.9 ppm) was estimated to be 6.3, 9.0, and 20.4, respectively (Supplementary Figure S1(b–d)).

The surface potential of the FA-Gd-Au PENPs (0.56 mV) after acetylation is close to neutral (Supplementary Table S1). UV-Vis spectroscopy was used to follow the PEI surface modification and to confirm the synthesis of AuNPs (Supplementary Figure S2). The PEI-mPEG-DOTA-(PEG-FA) displays two peaks at 286 and 361 nm, respectively which are related to the FA moieties. After the formation of FA-Gd-Au PENPs, a peak appearing at 549 nm can be assigned to a surface plasmon resonance (SPR) peak of AuNPs. These results indicate that FA-Gd-Au PENPs have been successfully prepared.

**Figure 2.**
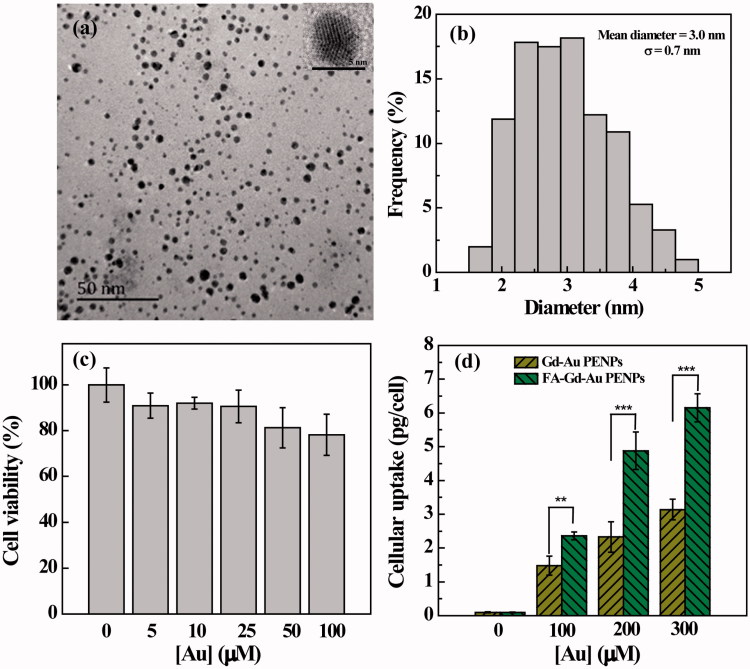
TEM image (a) and size distribution histogram (b) of the FA-Gd-Au PENPs. Inset of (a) is the high-resolution TEM image of a typical FA-Gd-Au PENP. (c) MTT assay of HeLa cell viability after treatment with the FA-Gd-Au PENPs at different Au concentrations for 24 h. Data are reported as mean ± SD (*n* = 3). (d) Cellular Au uptake in HeLa cells incubated with the FA-Gd-Au PENPs and nontargeted Gd-Au PENPs with different Au concentrations for 3 h.

The formed FA-Gd-Au PENPs were then characterized by TEM (Figure 2(a,b)). Clearly, the FA-Gd-Au PENPs display a uniform spherical morphology with a mean Au core diameter of 3 ± 0.7 nm. High-resolution TEM image of a typical FA-Gd-Au PENP shows the clear lattice structure, indicating that the Au core particles are crystalline. The hydrodynamic size of the FA-Gd-Au PENPs was further measured by DLS to be 188.9 nm, which is much larger than that measured by TEM (Supplementary Table S1). This is because DLS measures the clustered FA-Gd-Au PENPs in aqueous solution that may consist of many single AuNPs, whereas TEM only measures single Au core NPs in a dry state, in agreement with the literature (Zhou et al., 2016a,b). We also note that FA modification renders the FA-Gd-Au PENPs with a lower surface potential and a bigger hydrodynamic size than that of the non-targeted Gd-Au PENPs (Zhou et al., 2016a; Supplementary Table S1). This could be due to the fact that the negatively charged FA carboxyl groups lead to the lower surface potential of the final Au PENPs, and the modification of FA may promote slightly enhanced interparticle interaction to form bigger particle clusters in aqueous solution.

The stability of the formed FA-Gd-Au PENPs was also assessed by exposing them to water having different pHs and temperatures (Supplementary Figure S3). It can be seen that there is no significant change in the UV-Vis spectral features of the particles, particularly, the SPR peak of the AuNPs does not seem to have a change under the studied pH and temperature conditions. We also check the colloidal stability of the FA-Gd-Au PENPs dispersed in water, PBS, and cell culture medium. Clearly, the particles are quite colloidally stable after the solutions were stored at 4 °C for at least seven days. The good stability of the FA-Gd-Au PENPs are pre-requisite for their further biomedical applications.

### Cytotoxicity and cellular uptake assays

We used MTT assay to evaluate the cytotoxicity of the FA-Gd-Au PENPs (Figure 2(c)). It can be seen that HeLa cells treated with the FA-Gd-Au PENPs in an Au concentration range of 5–100 μM have approximately similar viability to the PBS control. The cytotoxicity of the FA-Gd-Au PENPs was further examined by observation of the morphology of cells treated with the particles (Supplementary Figure S4). We find that there are no apparent morphological changes occurring even at the Au concentration up to 100 μM in comparison to the control cells treated with PBS. These results indicated that the formed FA-Gd-Au PENPs have a good cytocompatibility in the given concentration range.

**Figure 3.**
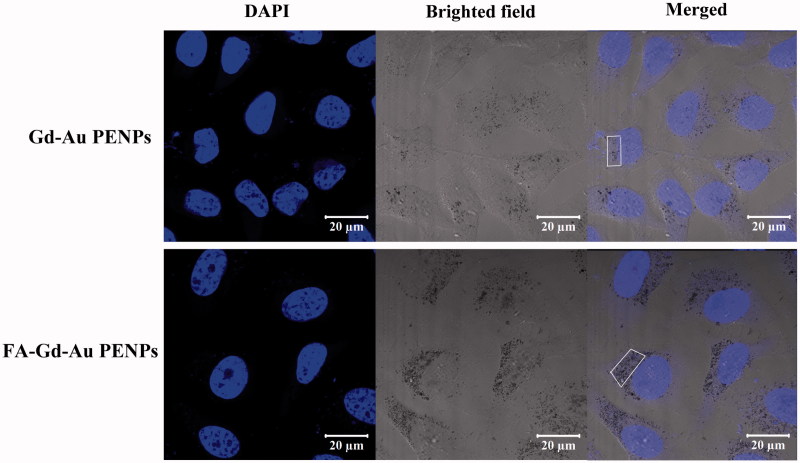
Confocal microscopic images of HeLa cells treated with the FA-Gd-Au PENPs and nontargeted Gd-Au PENPs ([Au] = 100 μM) for 12 h. The white frame refers to the cytophagic particles.

To investigate the FA-mediated specific cellular uptake, ICP-OES was used to quantify the Au uptake in HeLa cells treated with the FA-Gd-Au PENPs and nontargeted Gd-Au PENPs at different concentrations for 3 h. As shown in Figure 2(d), the Au uptake in HeLa cells treated with the FA-Gd-Au PENPs is much higher than that treated with the nontargeted Gd-Au PENPs (*p* < .001) under the same Au concentrations. In addition, we also note that HeLa cells treated with the FA-Gd-Au PENPs swallowed more particles than those treated with the nontargeted Gd-Au PENPs at the Au concentration of 100 μM from confocal microscopy (Figure 3, white frame). These results indicate that the designed FA-Gd-Au PENPs are able to target the FA receptor (FAR)-overexpressing cancer cells through a receptor-mediated pathway.

### X-ray attenuation property and T_1_ MR relaxometry of the FA-Gd-Au PENPs

**Figure 4.**
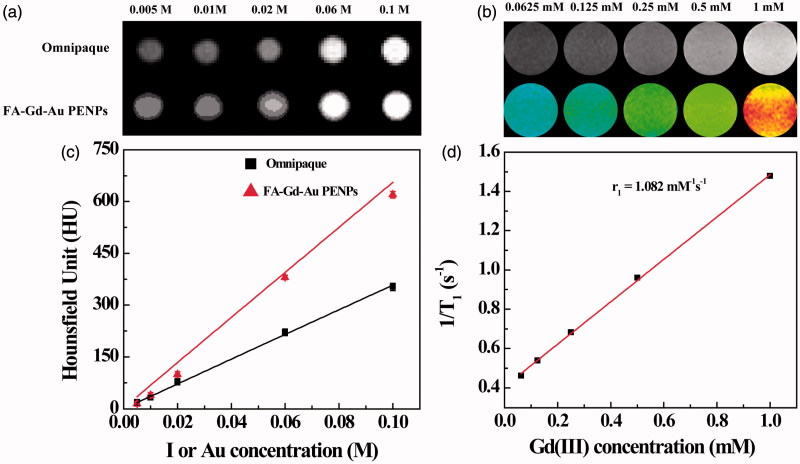
(a) CT phantom images and (c) X-ray attenuation intensity of the FA-Gd-Au PENPs and Omnipaque as a function of the molar concentration of the radiodense element (Au or iodine). (b) and (d) show the T_1_-weighted MR phantom images and the linear fitting of the inverse T_1_ of the FA-Gd-Au PENPs as a function of Gd concentration.

To examine the potential to use the developed FA-Gd-Au PENPs for CT and T_1_ MR imaging of tumors, we studied the X-ray attenuation property and T_1_ MR relaxometry of the particles (Figure 4). It is clear that the FA-Gd-Au PENPs and Omnipaque (as a control) display increased CT contrast enhancement with the increase of the imaging element (Au or iodine) concentration (Figure 4(a,c)). However, the CT value of the FA-Gd-Au PENPs is higher than that of Omnipaque at the same radiodense element concentration, especially at the high concentrations (Figure 4(c)), in agreement with our previous work (Zhou et al., 2014,2016a).

The presence of Gd(III) ions endowed the FA-Gd-Au PENPs with r_1_ relaxivity for MR imaging applications. MR phantom studies show that the MR signal intensity of the FA-Gd-Au PENPs increases with the Gd concentration in the MR images (Figure 4(b)). By linear fitting the inverse T_1_ as a function of Gd concentration, the r_1_ relaxivity of the FA-Gd-Au PENPs was measured to be 1.082 mM^−1^s^−1^ (Figure 4(d)), which is comparable to the nontargeted material reported in our previous work (Zhou et al., 2016a). Taken these results together, the designed FA-Gd-Au PENPs display both the enhanced X-ray attenuation property and reasonable r_1_ relaxivity, which is amenable for their dual mode CT/MR imaging applications.

### Targeted CT/MR dual mode imaging of tumors *in vivo*

**Figure 5.**
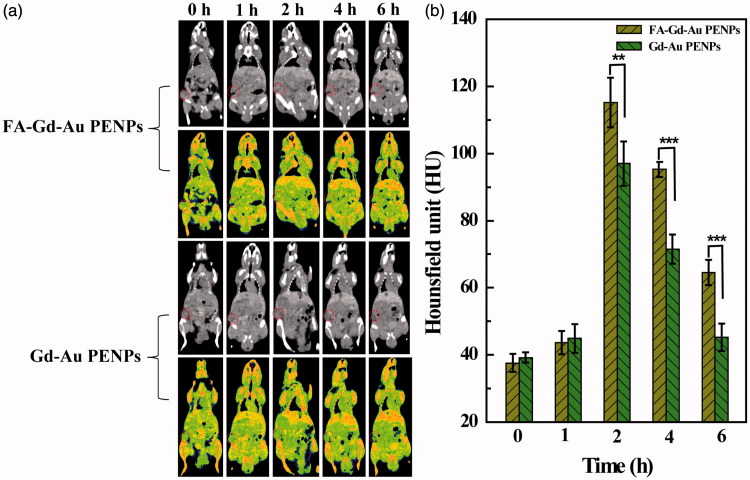
*In vivo* CT images (including renderings) (a) and CT values (b) of tumors at different time points post-injection of the FA-Gd-Au PENPs and nontargeted Gd-Au PENPs ([Au] = 0.1 M, 150 μL in PBS for each mouse). The circle in each panel refers to the tumors site.

We next used the designed FA-Gd-Au PENPs for CT imaging of tumors *in vivo*. As shown in Figure 5(a), the tumor region has an evident CT contrast enhancement after intravenous injection of the FA-Gd-Au PENPs or nontargeted Gd-Au PENPs, suggesting that the injected particles can be effectively transported to the tumor site likely via the passive EPR effect for both particles. At the same time points, the CT values of the tumor region treated with the FA-Gd-Au PENPs are much higher than those injected with the nontargeted Gd-Au PENPs (Figure 5(b)). At 4 h post-injection, the CT value of the tumor region treated with the FA-Gd-Au PENPs is 95.3 ± 2.3 HU, which is 25% higher than that treated with the nontargeted Gd-Au PENPs (*p* < .001). In addition, due to the metabolism of the particles, for both particles, maximum CT values are attained at 2 h post-injection and after 2 h post-injection the CT values of tumors decrease with time. Our results demonstrate that with the aid of FA-mediated active targeting, the formed FA-Gd-Au PENPs can have specifically enhanced particle accumulation in the tumor region, thereby leading to specific tumor CT imaging *in vivo*.

**Figure 6.**
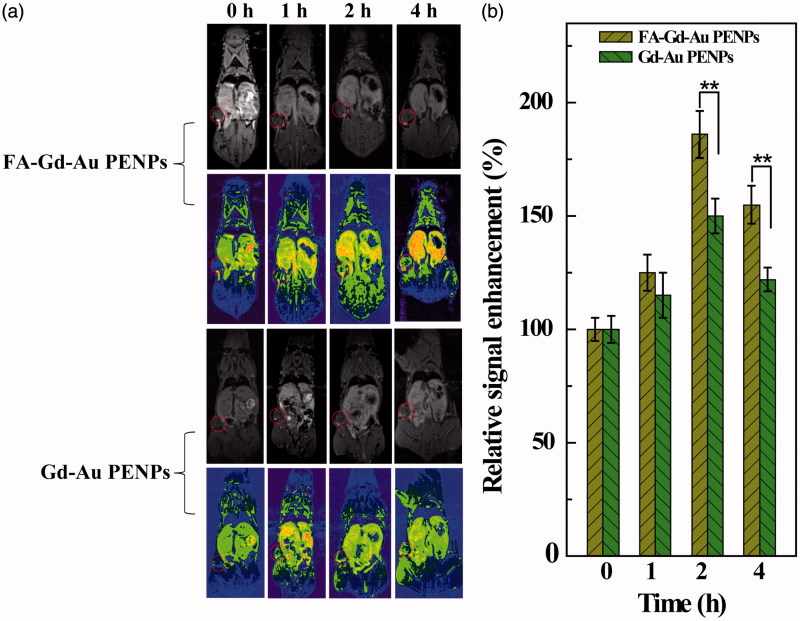
*In vivo* T_1_ *-*weighted MR images (including pseudo-color images) (a) and tumor signal enhancement percentage (b) of nude mice before and at different time points post-injection of the FA-Gd-Au PENPs and nontargeted Gd-Au PENPs ([Gd] = 0.01 M, 150 μL in PBS for each mouse). The circle in each panel refers to the tumor site.

We next checked the potential to use the formed FA-Gd-Au PENPs for targeted tumor MR imaging *in vivo* (Figure 6). It can be seen that the tumor region becomes brightened after injection of the FA-Gd-Au PENPs and nontargeted Gd-Au PENPs (Figure 6(a)). This also suggests that the FA-Gd-Au PENPs can be effectively delivered to tumor region, similar to the tumor CT imaging data. The MR signal enhancement percentage of the tumors treated with the FA-Gd-Au PENPs is much higher than that treated with the nontargeted Gd-Au PENPs at the same time points (Figure 6(b)). For instance, the MR signal enhancement percentage for the targeted group is 186 ± 10.4% at 2 h post-injection, which is significantly higher than that for the nontargeted group (150 ± 7.7%) at the same time point. Taken together, we can conclude that the designed FA-Gd-Au PENPs can be used as an effective nanoprobe for targeted CT/MR imaging of FAR-overexpressing tumors.

### *In vivo* biodistribution and H&E staining

To investigate the biodistribution of the Au PENPs, we analyzed the Au content in the major organs and tumor of the mice (Supplementary Figure S5). It can be seen that the particles are mainly phagocytosed by spleen, followed by liver, lung, and kidney. It is very common that PEGylated NPs are mainly cleared by the reticuloendothelial system (RES) and Kupffer cells of the liver and spleen, resulting in limited uptake in the tumor, in agreement with the literature (Kim et al., 2007). In any case, a portion of the FA-Gd-Au PENPs are able to escape the RES uptake, allowing for effective tumor CT/MR imaging.

To check the *in vivo* organ toxicity of the particles, we performed H&E staining of the major organs of mice after 30 days treatment with the particles (Supplementary Figure S6). Clearly, there are no histological changes observed in the major organs including liver, lung, spleen, kidney, and heart at one month post-injection, compared with the PBS control. This suggests that the designed FA-Gd-Au PENPs show good biocompatibility *in vivo*.

## Conclusion

In summary, we developed a facile approach in designing PEI-based FA-Gd-Au PENPs as a nanoprobe for targeted tumor CT/MR imaging. We show that the PEI is a powerful platform that can be linked with PEGylayted FA, entrapped with AuNPs with an Au core size of 3 nm, and chelated with Gd(III) ions. Moreover, the designed FA-Gd-Au PENPs are colloidally stable and cytocompatible in a given concentration range. Owing to the FA-mediated targeting specificity, as well as the enhanced X-ray attenuation property and reasonable r_1_ relaxivity, the FA-Gd-Au PENPs can be used as a dual mode nanoprobe for targeted tumor CT/MR imaging *in vivo*.

## Supplementary Material

IDRD_Shi_et_al_Supplemental_Content.doc
