# Supplementary material for: Targeted tumor dual mode CT/MR imaging using multifunctional polyethylenimine-entrapped gold nanoparticles loaded with gadolinium
Source: Drug Deliv. 2018 Jan 4;25(1):178–86. doi: 10.1080/10717544.2017.1422299 (PMC6058675; doi:10.1080/10717544.2017.1422299)
Supplement: IDRD_Shi_et_al_Supplemental_Content.doc [file IDRD_A_1422299_SM9831.doc]

**Supporting Information**

**Targeted tumor dual mode CT/MR imaging using multifunctional polyethylenimine-entrapped gold nanoparticles** **loaded with gadolinium**

Benqing Zhou^1,2^, Zuogang Xiong^1^, Peng Wang^2^, Chen Peng^1^*, Mingwu Shen^2^*, Serge Mignani^3,6^, Jean-Pierre Majoral^4,5^, Xiangyang Shi^1,2,6^*

^1^ Department of Radiology, Shanghai Tenth People’s Hospital, Tongji University School of Medicine, Shanghai 200072, P. R. China

^2^ State Key Laboratory for Modifcation of Chemical Fibers and Polymer Materials, College of Chemistry, Chemical Engineering and Biotechnology, Donghua University, Shanghai 201620, P. R. China

^3^ Université Paris Descartes, PRES Sorbonne Paris Cité, CNRS UMR 860, Laboratoire de Chimie et de Biochimie Pharmacologiques et Toxicologique, 45, rue des Saints Peres, 75006, Paris, France

^4^ Laboratoire de Chimie de Coordination du CNRS, 205 Route de Narbonne, BP 44099, 31077 Toulouse Cedex 4, France

^5^ Université de Toulouse, UPS, INPT, 31077 Toulouse Cedex 4, France

^6^ CQM - Centro de Química da Madeira, MMRG, Universidade da Madeira, Campus da Penteada, 9020-105 Funchal, Portugal

**Key words:** polyethylenimine; gold nanoparticles; CT imaging; MR imaging; tumor targeting

___________________________________________________________________________________________

* Corresponding author. E-mail address: [pengchen_1985@163.com](mailto:pengchen_1985@163.com) (C. Peng), [mwshen@dhu.edu.cn](mailto:mwshen@dhu.edu.cn) (M. Shen), and xshi@dhu.edu.cn (X. Shi)

[**Table S1**. Zeta potentials and hydrodynamic size of the FA-Gd-Au PENPs and Gd-Au PENPs. Data are presented as](app:ds:  quantitative analysis) Mean ± S.D. (n = 3).

| Samples | Zeta potential (mV) | Hydrodynamic size (nm) | Polydispersity index  (PDI) |
| --- | --- | --- | --- |
| FA-Gd-Au PENPs | 0.56 ± 0.02 | 188.9 ± 11.4 | 0.32 ± 0.02 |
| Gd-Au PENPs | 7.40 ± 1.00 | 81.1 ± 5.6 | 0.25 ± 0.03 |


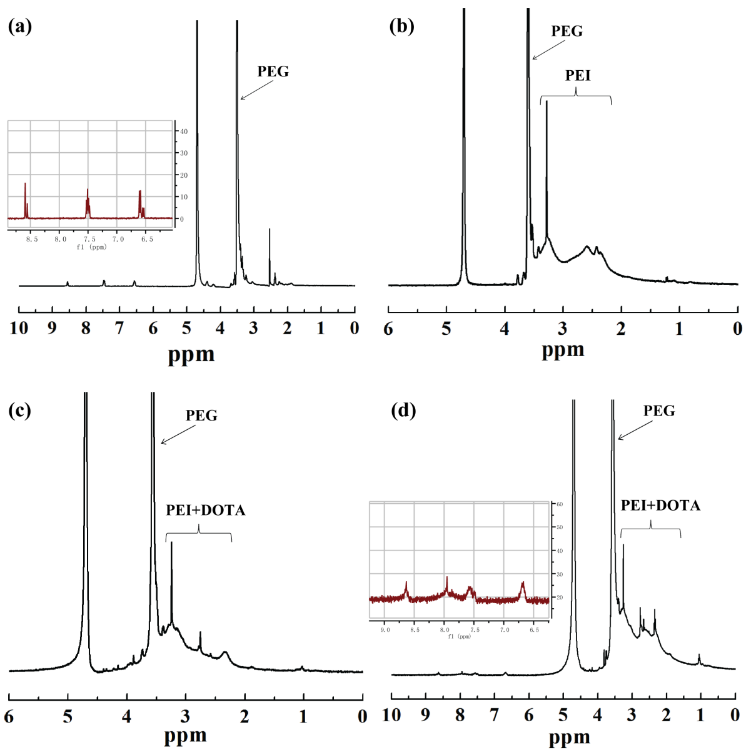


**Figure S1. ^1^**H NMR spectra of FA-PEG-COOH (a), PEI-*m*PEG (b), PEI-*m*PEG-DOTA (c), FA-Gd-Au PENPs (d), respectively.

**Figure S2.** UV-vis spectra of the PEI-*m*PEG-DOTA-(PEG-FA) and FA-Gd-Au PENPs.


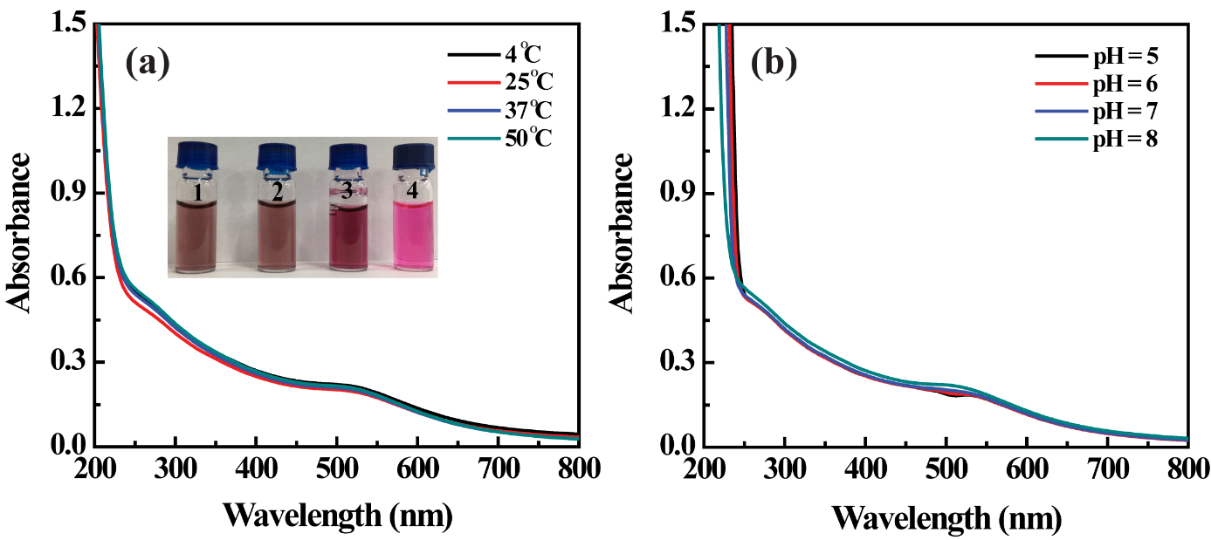


**Figure S3**. UV-vis spectra of the FA-Gd-Au PENPs solution at different temperatures (a) and pHs (b). Inset of (a) shows the particles dispersed in water (1), PBS (2), cell culture medium (3), respectively for 7 days. (4) is the blank cell culture medium.


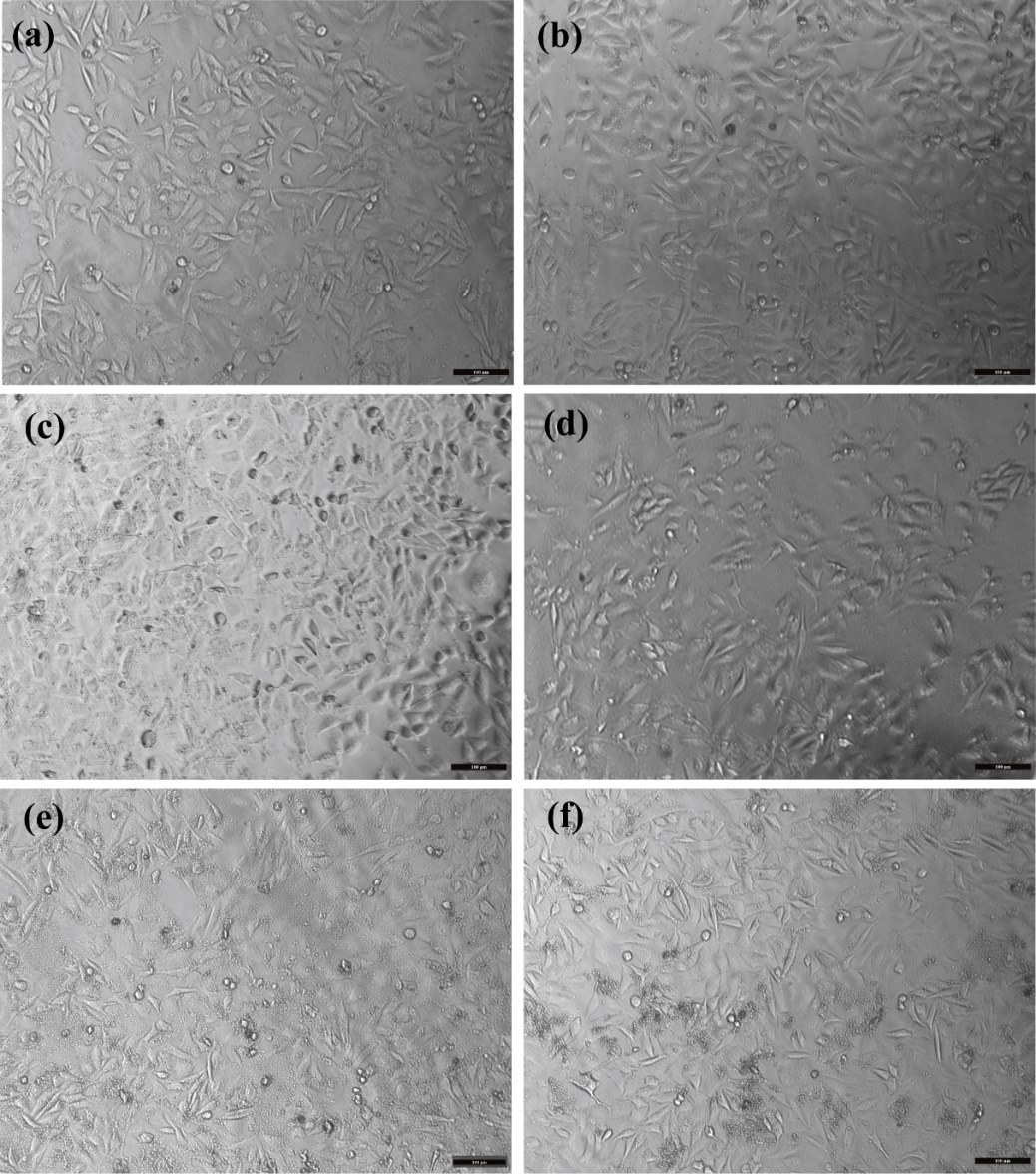


**Figure S4.** Micrographs of HeLa cells treated with PBS (a) and the FA-Gd-Au PENPs at the Au concentrations of 5 (b), 10 (c), 25 (d), 50 (e) and 100 (f) μM for 24 h, respectively. The scale bar in each panel represents 100 μm.

**Figure S5.** Biodistribution of Au in the major organs of the mice including heart, liver, spleen, lung, kidney, and tumor. The data were recorded from the whole organ or the whole tumor at different time points postinjection of the FA-Gd-Au PENPs ([Au] = 0.1 M, in 150 μL PBS for each mouse).

**Figure S6.** H&E staining of the sections of the heart, liver, spleen, lung, and kidney in healthy mice at 30 days post intravenous injection of the FA-Gd-Au PENPs ([Au] = 0.1 M, in 100 μL PBS for each mouse) or PBS (as a control). The scale bar in each panel represents 100 μm.
